# 布格替尼治疗非小细胞肺癌的研究新进展

**DOI:** 10.3779/j.issn.1009-3419.2025.106.12

**Published:** 2025-06-30

**Authors:** Jia YU, Shengxiang REN

**Affiliations:** 200082 上海，同济大学附属上海市肺科医院肿瘤科; Department of Oncology, Shanghai Pulmonary Hospital Affiliated to Tongji University, Shanghai 200082, China

**Keywords:** 肺肿瘤, 非小细胞肺癌, 间变性淋巴瘤激酶酪氨酸激酶抑制剂, 布格替尼, 疗效, 安全性, Lung neoplasms, Non-small cell lung cancer, Anaplastic lymphoma kinase-tyrosine kinase inhibitors, Brigatinib, Efficacy, Safety

## Abstract

随着间变性淋巴瘤激酶（anaplastic lymphoma kinase, *ALK*）酪氨酸激酶基因重排突变在非小细胞肺癌（non-small cell lung cancer, NSCLC）中被发现，针对ALK的靶向治疗不断发展。新一代ALK抑制剂布格替尼（Brigatinib）已在*ALK*阳性的NSCLC患者中被证明具有显著疗效，并在肿瘤深度缓解、脑转移患者的治疗、生活质量和长期生存等多方面均具有临床获益。本文将对布格替尼的最新研究进展和探索方向进行综述。

## Abstract (en)

The discovery of anaplastic lymphoma kinase (*ALK*) tyrosine kinase gene rearrangement mutations in non-small cell lung cancer (NSCLC) has driven continuous advancements in ALK-targeted therapies. The next generation of ALK tyrosine kinase inhibitor, Brigatinib, has demonstrated significant efficacy in patients with *ALK*-positive NSCLC, offering clinical benefits in deep response of tumor, treatment of brain metastases patients, quality of life, and long-term survival. This review will provide current advancements and exploratory directions for Brigatinib.

肺癌在全球癌症中的发病率和死亡率均位居第一^[1]^，其中非小细胞肺癌（non-small cell lung cancer, NSCLC）约占85%。随着靶向药物的开发，近年来在间变性淋巴瘤激酶（anaplastic lymphoma kinase, *ALK*）阳性NSCLC患者的治疗领域，ALK-酪氨酸激酶抑制剂（tyrosine kinase inhibitors, TKIs）发展迅速。NSCLC治疗的理想目标不仅需要使患者活得更长，也需使其活得更好，而ALK-TKIs应用中存在的耐药及脑转移患者预后差等问题，仍是长期以来的临床挑战。

新一代ALK-TKIs布格替尼（Brigatinib），已在*ALK*阳性局部晚期或转移性NSCLC患者的治疗中被证明具有良好的疗效与安全性，且是首个在生活质量方面具有显著获益的ALK-TKI。该药自2017年上市以来，进一步积累了循证证据。本文更新了“布格替尼的ALK肺癌试验（ALK in Lung Cancer Trial of Brigatinib, ALTA）”系列研究的最新结果以及多项布格替尼的真实世界研究和荟萃分析证据，梳理了该药于深度缓解、脑转移治疗、生活质量和长期生存等多维度的临床获益及其安全性管理，并展望其未来研究方向，以期为*ALK*阳性NSCLC患者的个体化治疗提供参考和启发。

## 1 布格替尼的概述

布格替尼是一种ALK以及表皮生长因子受体（epidermal growth factor receptor, EGFR）的可逆双重抑制剂，且对*ALK*、野生型*EGFR*、L858R/T790M耐药突变的*EGFR*、*c-ros*原癌基因1受体酪氨酸激酶（*c-ros* oncogene 1 receptor tyrosine kinase, ROS1）、FMS样酪氨酸激酶3（FMS-like tyrosine kinase 3, FLT3）以及胰岛素样生长因子1受体（insulin-like growth factor 1 receptor, IGF1R）等多种激酶的活性均有抑制作用^[2]^。该药可阻断ALK介导的下游JAK/STAT、MAPK或PI3K/AKT等信号通路，最终抑制肿瘤细胞增殖和生存^[3]^。

布格替尼的分子结构决定了其具有与铰链残基Met1199的氢键相互作用，与三磷酸腺苷结合口袋中关键残基Leu1122、Val1130、Leul198、Met1199、Gly1202、Leu1256和Asp1270的强疏水作用，以及与残基Lys1150的强静电相互作用^[4]^。基于这些结构特性，布格替尼相比第一代ALK-TKIs克唑替尼对ALK结合的亲和力更强，其在*ALK*阳性NSCLC细胞中对ALK的抑制活性高出克唑替尼12倍^[5]^。同时，该药对常见的17种*ALK*耐药突变体均具有选择性，对部分ALK耐药突变具有比其他ALK-TKIs更强的效力：在含有耐药相关棘皮动物微管相关蛋白样4（echinoderm microtubule-associated protein-like 4, *EML4*）-*ALK*突变体的NSCLC细胞中，布格替尼的抑制活性是克唑替尼的2.2-77倍，是塞瑞替尼/阿来替尼的≥3倍^[5]^。临床经验报道^[6-9]^显示，布格替尼可能克服L1152V/Q1146K突变介导的克唑替尼耐药、I1171S或L1196Q突变介导的阿来替尼耐药以及T1151R突变介导的塞瑞替尼耐药等，也可能对I1171S+G1269A复合突变具有敏感性^[10]^。此外，该药对中枢神经系统可能具有较好的渗透性，可显著提高脑肿瘤模型小鼠的生存期并减少小鼠脑部肿瘤负荷^[5]^。

布格替尼既往在I/II期临床研究中已初步显示出可安全、有效地治疗*ALK*基因重排的NSCLC患者，客观缓解率（objective response rate, ORR）可达75%，并确认了该药的推荐剂量为“前7天口服90 mg qd，然后增加剂量至180 mg qd”^[11]^。II期ALTA研究^[12]^中位随访8.0个月的结果显示，既往接受过克唑替尼治疗并发生疾病进展的*ALK*阳性局部晚期或转移性NSCLC患者，在接受上述推荐剂量的布格替尼治疗后ORR达到54%，中位无进展生存期（progression-free survival, PFS）为12.9个月。且大多数治疗周期患者的整体健康状况/生活质量评分均具有显著改善^[13]^。这些结果支持了布格替尼的进一步开发。

## 2 布格替尼在*ALK*阳性晚期NSCLC患者中的一线治疗

### 2.1 临床疗效评估

ALTA-1L研究是一项开放标签的III期随机对照试验，旨在比较布格替尼和克唑替尼对*ALK*阳性晚期NSCLC患者（既往未接受过ALK抑制剂治疗）的疗效与安全性，主要终点设计为盲态独立中心审查委员会（blinded independent review committee, BIRC）评估的PFS。2021年ALTA-1L研究的最终分析^[14]^（布格替尼中位随访40.4个月）显示，在275例意向治疗（intention-to-treat, ITT）患者人群中，无论是BIRC评估的[24.0 *vs* 11.1个月，风险比（hazard ratio, HR）=0.48，*P*<0.0001]还是研究者评估的（30.8 *vs* 9.2个月，HR=0.43，*P*<0.0001）结果，接受布格替尼治疗患者的中位PFS均显著优于克唑替尼治疗组。在总生存期（overall survival, OS）结局方面，两组患者的4年OS率分别为66%和60%；进一步调整去除克唑替尼组交叉因素后，布格替尼较克唑替尼可降低死亡风险达46%^[14]^。最终ORR结果与既往中期分析一致，值得注意的是，两组达到BIRC评估的完全缓解的患者比例分别为24%和13%；且布格替尼组达到深度缓解（定义为靶病灶较基线缩减76%-100%）的患者比例明显更高（56% *vs* 34%, *P*=0.0005），实现深度缓解患者的中位PFS可达44个月（HR=0.23），中位OS尚未达到（HR=0.15）^[15]^。为了最大限度地减少或消除残留病变，避免耐药发生，且基于ALTA-1L研究中布格替尼较高的深度缓解率，BRIGHTSTAR研究^[16]^设计将该药诱导治疗联合局部巩固治疗方案（手术或放疗）用于*ALK*重排的晚期NSCLC患者，初步结果显示布格替尼诱导治疗带来的患者疾病控制率（disease control rate, DCR）为100%，ORR达到79%，62%的患者接受了完全的局部巩固治疗，联合局部巩固治疗1、2和3年后的PFS率分别为94%、80%和66%，提示布格替尼可为*ALK*阳性晚期NSCLC患者争取局部巩固治疗的机会，以进一步延长生存时间。

在ALTA-1L研究^[14]^中，BIRC分别评估了ITT人群和基线伴脑转移人群的颅内中位PFS。ITT人群中布格替尼组患者的颅内中位PFS可达到44.1个月（克唑替尼组21.2个月，*P*<0.0001）。对于任意基线脑转移患者，布格替尼组颅内中位PFS（24.0个月）明显长于克唑替尼组（5.5个月，HR=0.29，*P*<0.0001），4年OS率也明显更高（71% *vs* 44%, *P*=0.020）；颅内ORR分别为66%和14%（*P*<0.0001），其中颅内完全缓解比例分别为45%和2%^[14]^。部分患者在基线时具有可测量的脑转移病灶，在这些人群中布格替尼组的颅内ORR也可达78%（克唑替尼组为26%，*P*=0.0014），颅内完全缓解比例分别为28%和0%^[14]^。

另一项基于ALTA-1L研究的亚组分析^[17]^显示，布格替尼在BIRC评估的中位PFS方面，108例亚洲（24.0 *vs* 11.1个月，HR=0.35，*P*=0.0001）和167例非亚洲（24.7 *vs* 9.4个月，HR=0.56，*P*=0.0041）患者之间是一致的，均优于克唑替尼。

近期一项针对*ALK*重排NSCLC患者的回顾性研究^[18]^发现，在真实世界中接受布格替尼作为一线治疗（32例）后ORR和颅内ORR可分别达93.8%和100%，12个月PFS率和OS率分别为84.1%和95.2%，进一步支持布格替尼作为一线治疗方案具有良好的疗效。

此外，多项将ALK抑制剂作为*ALK*阳性晚期NSCLC患者一线治疗的荟萃分析/系统性综述发现了以下结果：（1）布格替尼与克唑替尼、塞瑞替尼相比显著延长了BIRC评估的中位PFS；在OS方面，布格替尼与塞瑞替尼、恩沙替尼、阿来替尼或洛拉替尼的疗效无明显差异^[19]^。（2）在几项伴有中枢神经系统转移患者的研究中，以克唑替尼为共同对照进行间接比较发现，布格替尼的PFS累积排序概率曲线下面积（surface under the cumulative ranking curve, SUCRA）（95.3%）高于阿来替尼（54.8%）和克唑替尼（0%）^[20]^；布格替尼在6种治疗方案（洛拉替尼、布格替尼、阿来替尼、塞瑞替尼、克唑替尼和铂类化疗）分析中的PFS位列第二位，SUCRA为88.0%，且与SUCRA首位的洛拉替尼（88.3%）的疗效无统计学差异^[21]^；另一项多种治疗方案（紫杉醇类化疗、铂类化疗以及布格替尼、克唑替尼、阿来替尼、塞瑞替尼、洛拉替尼）分析^[22]^中，布格替尼的ORR最佳，SUCRA为0.824。（3）在亚洲患者人群中，使用洛拉替尼和布格替尼治疗的PFS可能无显著差异^[21]^，也有荟萃分析^[23]^提示布格替尼组具有最高的PFS获益排名，其SUCRA值（47%）高于阿来替尼（35%）、洛拉替尼（17%）、塞瑞替尼（0%）和克唑替尼（0%）组。

综上可见，布格替尼作为一线治疗可以延长全人群的PFS和OS，实现可观的深度缓解，不仅对于脑转移患者具有良好疗效，还可能为晚期患者带来局部巩固治疗的机会，且该药广泛适用于亚洲和非亚洲人群。

### 2.2 生活质量获益

克唑替尼或塞瑞替尼曾被证明相对化疗可改善生活质量，在ALK抑制剂之间的比较中，阿来替尼、恩沙替尼和洛拉替尼均未获得生活质量方面的理想获益^[24-28]^。而布格替尼是目前报告的唯一能够延长患者生活质量恶化时间的ALK-TKIs。在所有ALK-TKIs中布格替尼是首个报告其健康相关生活质量获益优于另一种ALK抑制剂的药物，包括对认知功能、情感功能、社交功能等的改善，以及对疲劳、胃肠道症状（恶心和呕吐、食欲降低、便秘）的缓解^[29]^。

ALTA-1L研究^[29]^结果发现，布格替尼和克唑替尼组整体健康状况/生活质量评分恶化的中位时间分别为26.74和8.31个月（*P*=0.0485）。相较于对克唑替尼治疗有应答患者，布格替尼应答患者的健康相关生活质量恶化时间进一步延迟（37.9 *vs* 13.0个月，*P*=0.1225），提示布格替尼一线治疗应答患者的生活质量更高^[30]^。一项基于ALTA-1L研究的分析^[31]^显示，与克唑替尼相比，接受布格替尼治疗效果显著且具有临床意义地提高了BIRC（*P*=0.045）及研究者评估（*P*=0.018）的质量调整的无疾病或毒性症状生存时间，支持该药作为一线疗法可使患者达到更理想的生存状态。有荟萃分析^[32]^结果进一步表明，在治疗*ALK*阳性晚期NSCLC患者的一线方案中（化疗或ALK抑制剂），布格替尼在生活质量评分方面的结果表现最佳。

## 3 布格替尼在*ALK*阳性晚期NSCLC患者中的二线及后线治疗

布格替尼I/II期研究（137例）及II期ALTA研究（222例）的长期结局分析^[33]^报告了该药在克唑替尼经治局部晚期或转移性*ALK*阳性NSCLC患者中的显著疗效，中位PFS和OS分别达到了16.7和40.6个月，5年OS率为42%；且在基线伴任意脑转移患者中布格替尼组颅内中位PFS可达18.4个月，2年颅内PFS率为33%。III期ALTA-3研究^[34]^也纳入了248例克唑替尼经治的*ALK*阳性晚期NSCLC患者，结果显示布格替尼和阿来替尼组患者的BIRC评估的中位PFS获益相当，且安全性结果与既往报道一致。

在另一项单臂设计的ALTA-2研究^[35]^中，103例*ALK*阳性NSCLC患者经新一代ALK-TKIs阿来替尼或塞瑞替尼治疗进展后使用布格替尼治疗，由独立审查委员会（independent review committee, IRC）评估的ORR为26.2%，中位持续缓解时间为6.3个月，中位PFS为3.8个月。来自日本J-ALTA研究^[36]^的最终分析显示，在接受过阿来替尼±克唑替尼治疗的人群中（47例），布格替尼治疗后经IRC评估的ORR为34%，DCR为79%，PFS达7.3个月，中位OS达28个月；在32例非ALK抑制剂经治队列中，布格替尼治疗后IRC评估的ORR为97%，2年PFS率为73%。布格替尼在该研究中无论作为一线或后线治疗，在*ALK*阳性NSCLC患者中均显示出具有临床意义的疗效。基于ALTA-2和J-ALTA研究的合并分析^[37]^显示，阿来替尼经治的133例患者使用布格替尼治疗后，中位PFS为5.2个月，中位OS未达到，ORR为30.8%，进一步支持布格替尼作为后线治疗的获益潜能。

在近年来的真实世界环境中，一项中国单中心研究^[38]^显示，104例克唑替尼经治的*ALK*阳性NSCLC患者，接受布格替尼、阿来替尼和塞瑞替尼治疗的中位OS分别为43.8、34.5和19.4个月，组间中位生存结局无显著差异；但在克唑替尼耐药的脑转移患者中，布格替尼（66.7%）和阿来替尼（72.4%）的ORR显著高于塞瑞替尼（50.0%）和恩沙替尼（40.0%）。另一项韩国研究^[39]^报道显示，174例克唑替尼难治性*ALK*阳性NSCLC患者在接受布格替尼治疗后的2年OS率为68.7%。一项全球多中心真实世界研究^[40]^纳入了604例既往接受过洛拉替尼、塞瑞替尼、阿来替尼或克唑替尼治疗的*ALK*阳性NSCLC患者，其服用布格替尼的中位治疗终止时间分别为7.5、10.3、8.7和10.0个月，提示无论既往ALK-TKIs的使用情况如何，在临床实践中应用布格替尼均可能有效且可耐受。

## 4 布格替尼的安全性

ALTA-1L研究^[14]^中布格替尼常见的治疗期间发生的不良事件（adverse events, AEs）包括腹泻（58%）、血肌酸磷酸激酶水平升高（50%）、咳嗽（36%）、恶心（33%）、高血压（32%）、天冬氨酸氨基转移酶水平升高（26%）、脂肪酶升高（24%）和丙氨酸氨基转移酶水平升高（23%）等；≥3级AEs最常见为血肌酸磷酸激酶升高（26%）、脂肪酶升高（15%）、高血压（14%）、淀粉酶升高（6%）、肺炎（5%）等。荟萃分析^[41]^结果显示，在6种ALK-TKIs的1-5级AEs的累积排名曲线中，布格替尼累积曲线下面积最小，提示其具有良好的安全性。

布格替尼需要关注的AEs包括间质性肺病（interstitial lung disease, ILD）/非感染性肺炎，长期结局报告的该药ILD/非感染性肺炎发生率为6%^[14]^。肺部毒性反应是所有TKIs类药物的AEs之一，虽然发生率低，但临床实践中仍需重视并积极监测。ALK-TKIs总体上均具有可耐受的肺毒性，荟萃分析^[42]^显示布格替尼与全级别（7.09%）和高级别肺炎（3.06%）的发病率相关，未报告5级肺炎发生，因此需尽早发现和治疗肺炎，防止其恶化。一项药品上市后AEs信号分析^[43]^发现，克唑替尼、阿来替尼、塞瑞替尼、布格替尼和洛拉替尼治疗相关ILD的发生例数分别为160、75、22、10和4例，提示真实世界中使用这几种ALK-TKIs均可能发生ILD；另一项药物警戒分析^[44]^也得出类似结果，且布格替尼、克唑替尼、阿来替尼、洛拉替尼和塞瑞替尼组的ILD中位发病时间依次为4.5、25、35.5、54.5和84 d，可见布格替尼相关ILD出现较早。布格替尼治疗期间报告的肺毒性反应被定义为早发性肺事件（early-onset pulmonary event, EOPE），总发生率约为4.3%，具有发生时间早、发生率与起始剂量有关、患者可快速耐受以及症状可逆等特征^[45]^。

不同ALK-TKIs相关的AEs谱存在差异。在真实临床环境中，基于美国食品药品监督管理局AEs报告系统数据的药物警戒分析发现，不同ALK-TKIs报告的安全性信号可见于不同的系统和器官：布格替尼主要为呼吸系统疾病[报告比值比（reporting odds ratio, ROR）=2.31]，克唑替尼为眼部疾病（ROR=2.12），阿来替尼为肝胆疾病（ROR=2.63），塞瑞替尼为胃肠道疾病（ROR=2.41）、肝胆疾病（ROR=4.52）和呼吸系统疾病（ROR=2.08），洛拉替尼为代谢紊乱（血甘油三脂升高ROR=198.79）以及神经系统毒性[如记忆障碍（ROR=6.79）、神经混乱状态（ROR=5.17）、周围神经病变（ROR=4.87）、触觉减退（ROR=4.86）等]^[43,46]^。在针对ALK-TKIs心脏毒性的药物警戒分析^[47]^中，洛拉替尼和阿来替尼显示出阳性信号，而布格替尼未显示任何阳性信号；针对ALK-TKIs肝脏毒性的药物警戒分析^[48]^显示，塞瑞替尼的丙氨酸氨基转移酶升高和天冬氨酸氨基转移酶的发生率在6种ALK-TKIs中最高（49.72%和43.20%），而布格替尼组发生率相对较低（15.32%和16.71%）。

由中国临床肿瘤学会（Chinese Society of Clinical Oncology, CSCO）非小细胞肺癌专业委员会组织编写的2023版《ALK-TKI药物不良反应管理专家共识》^[49]^，杨明意教授等发表的《布格替尼治疗*ALK*阳性NSCLC患者期间相关早发性肺事件及其管理策略》^[45]^，以及《间变性淋巴瘤激酶酪氨酸激酶抑制剂治疗晚期非小细胞肺癌中国专家建议（2024版）》^[50]^中，均提供了一些针对ALK-TKIs AEs的建议，包括针对消化道症状、肝肾损伤、水肿、肺毒性、心脏毒性等AEs的分级处理。在肺部毒性反应管理方面，由于布格替尼带来的EOPE可在持续用药期间迅速耐受，因此需要加强教育、采用适当的支持性护理和用药以维持治疗。所有接受布格替尼治疗的患者均应监测是否出现肺部受损的呼吸道症状，同时排查其他可能导致肺炎的原因。发生1或2级EOPE的患者，建议严格按照布格替尼说明书进行恰当的剂量调整、监测临床表现和实验室报告变化、积极采取对症支持治疗等有效管理手段；出现3或4级EOPE时应永久性终止治疗^[45]^。

## 5 布格替尼耐药问题的探讨

使用ALK-TKIs的NSCLC患者最终均可能发生耐药，其机制中获得性耐药相比固有耐药更为常见，并可进一步分为ALK依赖型和ALK非依赖型耐药（如旁路信号机制）。新一代ALK-TKIs的主要耐药机制为ALK依赖型突变机制，约占56.8%，这些突变可导致ALK蛋白中产生构象变化，从而干扰其与ALK抑制剂的结合；ALK非依赖型耐药机制约占18.2%[如B-Raf原癌基因（B-Raf proto-oncogene, *BRAF*）、Kirsten大鼠肉瘤病毒致癌基因同源物（Kirsten rat sarcoma viral oncogene homolog, *KRAS*）基因突变和间质表皮转化因子（mesenchymal to epithelial transition factor, *MET*）扩增等]，其他不确定的耐药机制约占25%^[51]^。目前已知的布格替尼ALK依赖型突变机制包括单点突变（如G1202R、D1203N、G1206C等）、含G1202R的复合突变以及其他复合突变（如T1151R+C1156Y+E1161D+F1174L）；布格替尼ALK非依赖型耐药机制可能涉及*mTOR*^T1834-T1837del^突变、*JAK3*^R948C^突变、*CDKN2A/B*功能缺失突变、*NFE2L2*^E79Q^突变、*MET*扩增、BRAF^V600E^和KRAS^G12D^突变等^[52]^。有研究^[4]^发现L1196M、G1269A、F1174L和R1275Q突变可引起布格替尼的多种构象变化以及ALK残基Glu1167、Arg1209、Asp1270和Asp1203的明显能量变化，可能导致耐药发生。Diaz-Jimenez等的研究^[53]^还发现布格替尼产生耐药的部分机制也可能与Src原癌基因酪氨酸蛋白激酶表达上调有关。

布格替尼最常见的单点耐药突变类型包括G1202R（43%）、E1210K（29%）、D1203N（14%）和S1206Y/C（14%）^[54]^。在已知的几种ALK-TKIs中，针对最常见的G1202R突变，洛拉替尼和伊鲁阿克的半抑制浓度（half inhibitory concentration, IC_50_）分别为4.99×10^-8^和9.6×10^-8^ mol/L，相比第一代/其他新一代ALK-TKIs均更低，理论上支持其成为G1202R突变的布格替尼（IC_50_=1.295×10^-7^ mol/L）耐药患者的后线选择；类似地，基于IC_50_结果，在布格替尼耐药患者中洛拉替尼或阿来替尼可能作为D1203N突变患者的序贯治疗，E1210K突变患者则可考虑选择洛拉替尼或塞瑞替尼，S1206Y突变患者或可考虑阿来替尼或塞瑞替尼^[54-56]^。复合突变在新一代ALK-TKIs耐药患者中比例较低，如F1174L+G1202R（4.5%）和L1196M+G1202R（2.3%）；接受布格替尼治疗的患者还可能存在D1203N+E1210K和D1203N+F1174C等复合耐药突变^[51,54,57]^。基于IC_50_结果，存在D1203N+F1174C复合突变的布格替尼耐药患者可考虑序贯洛拉替尼或阿来替尼，D1203N+E1210K复合突变的患者可考虑洛拉替尼、阿来替尼或塞瑞替尼^[54]^。

但上述布格替尼耐药后的治疗方案仍需要更多临床研究证据和真实世界经验的验证，目前美国国立综合癌症网络（National Comprehensive Cancer Network, NCCN）的NSCLC指南仅推荐了洛拉替尼作为G1202R和L1196M耐药突变后的可选ALK-TKIs^[58]^。Delmonte等^[59]^的真实世界研究报告显示，在布格替尼一线治疗进展后大多数*ALK*阳性NSCLC患者使用了另一种ALK-TKI，并获得了长期临床获益，全程中位PFS为51.6个月；其中一线布格替尼序贯二线洛拉替尼治疗患者的ORR和DCR分别为30.8%和76.9%，全程中位PFS可达74.7个月。BrigAlec研究^[60]^发现，接受布格替尼治疗的*ALK*阳性转移性NSCLC患者，在因AEs停药（中位PFS为10.9个月）后开始阿来替尼治疗，此后的中位PFS和OS分别为4.8和27个月。近期Wu等^[51]^的回顾性分析发现，既往接受过新一代ALK-TKIs治疗失败后选择化疗的*ALK*阳性晚期NSCLC患者，中位PFS和OS分别为6.9和16.3个月，提示对于身体状况较好的患者，化疗也可以作为布格替尼耐药后的一种选择。目前尚缺乏布格替尼联合抗血管靶向药物的临床研究，阿来替尼联合贝伐珠单抗的I/II期试验初步数据^[61]^或值得参考，该联合方案可使ALK-TKIs经治的晚期ALK重排NSCLC患者的中位PFS达到9.5个月。Yang等^[62]^还报告了1例*ALK*阳性肺鳞状细胞癌患者，在克唑替尼、阿来替尼先后耐药后接受了免疫治疗，实现了PFS超过29个月并获得了临床完全缓解，这也可为布格替尼序贯免疫治疗方案提供启发。

总体上，经布格替尼治疗后进展的患者，临床医生可根据药物对ALK特异性突变的敏感性更换其他合适的ALK-TKIs，也可考虑选择化疗、联合方案（联合靶向治疗或免疫治疗）或参加临床试验等。针对旁路耐药突变的患者，联合旁路抑制剂也可能是有利的治疗方案。

## 6 布格替尼的经济学评估

近期一项中国研究^[63]^基于马尔可夫模型对CSCO指南推荐的6种*ALK*阳性NSCLC患者的一线ALK-TKIs进行了成本效果分析，发现阿来替尼（187,143美元）和洛拉替尼（178,000美元）成本较高，与恩沙替尼（153,642美元）相比不具有成本效果；而在较恩沙替尼具有成本效果的布格替尼（156,314美元）和塞瑞替尼（147,216美元）之中，布格替尼的质量调整生命年更高（分别为3.67和3.00年），该研究提示在上述药物中布格替尼是最具成本效果的治疗方案。另一项基于中国医疗系统数据的药物经济学研究^[64]^显示，在成本最小化分析中，与阿来替尼相比，布格替尼可节约24,574美元的成本；在成本效果分析中，布格替尼相比阿来替尼获得了0.29个增量质量调整生命年，且成本也更低（-22,710美元）。因此，布格替尼可能是目前中国*ALK*阳性NSCLC一线治疗中最具成本效果的ALK-TKIs。

## 7 正在进行中的布格替尼相关临床研究

目前已有研究开始探索布格替尼能否作为新辅助治疗方案，包括在术前可切除的*ALK*阳性NSCLC患者中评估使用该药后的耐受持续状态的时间窗或生存预后（NCT05361564）。在将布格替尼作为*ALK*阳性NSCLC患者一线单药治疗的探索中，Brilliant研究（NCT05721950）、ABRAID研究（UMIN000042439）和ENTIRETY研究（NCT05735327）将分别在真实世界的中国、日本和波兰人群中评价该药的疗效与安全性；单臂II期B3i试验（NCT04634110）将在伴有脑转移的患者中评估布格替尼单药治疗效果。此外，CUBIK研究（NCT04223596）还将探索液体活检作为布格替尼疗效评估方法的实用性。

未来布格替尼与不同机制药物的联合治疗也可能为*ALK*阳性NSCLC患者的治疗带来新思路，如联合抗血管药物、化疗药物、免疫药物和神经类药物（β2受体阻滞剂、抗癫痫类药物）等。目前进入临床试验阶段的MASTERPROTOCOL ALK研究（NCT05200481）正在探索布格替尼联合卡铂+培美曲塞方案作为*ALK*阳性晚期NSCLC患者一线治疗的安全性和有效性；布格替尼联合贝伐珠单抗（NCT04227028）的I期研究也正在经治的*ALK*阳性NSCLC患者中积极开展。

在联合放疗的探索方面，单臂设计的干预性研究BRIGHTSTAR研究^[16]^纳入34例既往未接受过TKIs治疗的ALK重排转移性NSCLC患者（82%的患者基线时存在>3个肿瘤病灶），在接受8周的布格替尼的诱导治疗后接受放疗（27例）、手术（3例）或放疗+手术（2例）开展的局部巩固治疗，其中19例患者在放疗期间使用了布格替尼。结果显示，布格替尼联合局部巩固治疗的3年PFS率为66%（ATLA 1L研究中布格替尼3年PFS率为47%），≥3级与局部巩固治疗相关的AEs包括恶心、呕吐、食管炎、肺炎、贫血和支气管肺出血，未发生与局部巩固治疗相关的5级AEs。该研究提示布格替尼联合放疗对上述患者可能是一种安全可行的治疗方案，也期待进一步证据的补充和验证。

布格替尼对多种激酶均具有抑制作用，因此近年来也有多项小样本量研究或病例报告针对ALK外的其他靶点进行了探索。除ALK外，布格替尼同时也是EGFR抑制剂，伴EGFR ex19del/T790M/cis-C797S突变的NSCLC患者经奥希替尼治疗发生耐药后，联合（奥希替尼、西妥昔单抗）或单独使用布格替尼或许可能是一种有效、可行的方案^[65,66]^。由于布格替尼可通过抑制ST3GAL4/MET信号通路使耐药细胞对奥希替尼重新敏感，奥希替尼联合布格替尼可能是克服因ST3GAL4过表达和*MET*扩增导致奥希替尼耐药的有效策略^[67]^。近期，Niho等^[68]^的II期研究结果显示，布格替尼可有效治疗未经ALK-TKIs治疗的ROS1重排NSCLC患者，ORR达71.4%，PFS为12个月，且该药对克唑替尼耐药的ROS1重排NSCLC患者也具有一定的疗效。对于转移性*ALK*阳性NSCLC，由于布格替尼能同时靶向MARK2/3，该药可能更有效地抑制NSCLC细胞的迁移^[69]^。

除NSCLC之外，布格替尼也探索了其他瘤种人群的治疗方向，包括儿童/青少年*ALK*阳性间变性大细胞淋巴瘤/炎性肌纤维母细胞瘤/其他实体瘤（NCT04925609），以及神经纤维瘤病2型相关的进行性神经鞘瘤病^[70]^。临床前研究提示，该药在结直肠癌^[71]^、*ALK*阳性神经母细胞瘤^[72]^等肿瘤中也可能具有获益潜能。

## 8 总结和展望

基于ALTA系列及真实世界研究的循证证据，在*ALK*阳性NSCLC患者中新一代ALK-TKIs布格替尼已被证明具有良好的疗效和安全性，且适用于亚洲人群；该药对于脑转移患者具有明显生存获益优势，在改善患者生活质量和实现深度缓解等方面也显示出可观的应用前景。在2024年NCCN及2023年CSCO公布的NSCLC诊疗指南中，布格替尼均已被推荐为*ALK*阳性晚期NSCLC患者的一线优选治疗方案^[58,73]^。

安全性和耐药问题是ALK-TKIs未来需要继续攻克的挑战。目前已知的布格替尼常见AEs和可能出现的EOPE是可控的，可根据相关指南/共识的指导意见进行及时处理；在布格替尼耐药后可考虑使用洛拉替尼，或更换其他新一代ALK-TKIs。但针对布格替尼特定AEs的管理策略和针对其耐药性的方案选择及优化，仍需要不断研究探索和完善，从而进一步提高患者的治疗依从性和生活质量。近年来，从作为新辅助/辅助治疗方案、一线单药到作为各种联合方案的药物选择，已有大量布格替尼相关临床试验正在积极探索，以期实现其更广泛的临床应用。此外，针对布格替尼的经济学评估也仍需继续研究，目前该药已进入中国医保目录，有望进一步提高成本效果，为更多患者带来治疗获益。
